# Identification and Genomic Characterization of Bovine *Boosepivirus* A in the United States and Canada

**DOI:** 10.3390/v16020307

**Published:** 2024-02-17

**Authors:** Christian Savard, Leyi Wang

**Affiliations:** 1 Biovet Inc., 4375, Avenue Beaudry, Saint-Hyacinthe, QC J2S 8W2, Canada; christian.savard@biovet-inc.com; 2 Veterinary Diagnostic Laboratory, Department of Veterinary Clinical Medicine, College of Veterinary Medicine, University of Illinois, Urbana, IL 61802, USA

**Keywords:** *Boosepivirus* A, cattle, identification, genomic characterization

## Abstract

*Boosepivirus* is a new genus in the *Picornaviridae* family. Boosepiviruses (BooVs) are genetically classified into three species: A, B, and C. Initially, *Boosepivirus* A and B were identified in cattle, whereas *Boosepivirus* C was detected in sheep. Recent evidence showed that *Boosepivirus* B was detected in sheep and *Boosepivirus* C was identified in goats, suggesting that *Boosepvirus* might cross the species barrier to infect different hosts. Different from BooV B, BooV A is less studied. In the present study, we reported identification of two North American BooV A strains from cattle. Genomic characterization revealed that US IL33712 (GenBank accession #PP035161) and Canada 1087562 (GenBank accession #PP035162) BooV A strains are distantly related to each other, and US IL33712 is more closely correlated to two Asian BooV A strains. US-strain-specific insertions, NorthAmerican-strain-specific insertions, and species A-specific insertions are observed and could contribute to viral pathogenicity and host adaptation. Our findings highlight the importance of continued surveillance of BooV A in animals.

## 1. Introduction

Picornaviruses are small, nonenveloped icosahedral viruses containing a single-stranded positive-sense genome with sizes ranging from 6700 to 10,100 nucleotides. Picornaviruses have similar genome organizations with the 5′ end linked to a small peptide (VPg), a poly(A) tail at the 3′ end, and a single open reading frame flanked by untranslated regions (UTRs) at both ends [1]. Picornaviruses have an icosahedral capsid consisting of 60 copies of four structural proteins, VP1, VP2, VP3, and VP4. It has been reported that capsid proteins of picornaviruses are responsible for interacting with the host receptor and contributing to viral antigenicity and pathogenicity [2,3]. As of 26 December 2023, the *Picornaviridae* family has 68 proposed genera and 158 species at the International Committee on Taxonomy of Viruses. The *Boosepivirus* is a recently proposed genus with its name derived from bovine, ovine sapelo-entero-like picornavirus [4,5]. Based on the species demarcation criteria (≥30% divergent in polyprotein aa sequence; ≥40% divergent in P1 aa sequence; ≥35% divergent in 2C + 3CD sequence), the *Boosepivirus* genus is classified into three species: A, B, and C [6].

Bovine boosepivirus (BooV) was first identified in Japan on 5 of 40 fecal samples collected in diarrheic cattle. One sample sequence was classified as *Boosepivirus A*, and the remaining four were classified as *Boosepivirus B* [7]. This study suggested that bovine BooV might contribute to enteric diseases in cattle. Following the initial identification in Japan, bovine BooV B was then detected in different US states, and all the BooV positive samples had coinfection with other diarrheal pathogens [4]. Another study also revealed that bovine BooV B was detected in 64 of 603 samples collected from 20 provinces in China, and coinfection was present in 35 of these BooV positive samples [8]. Different from bovine BooV A and B, *Boosepivirus* C was initially detected in the cerebellum and spinal cord of sheep with severe encephalomyelitis in the UK and was revealed as a neuroinvasive picornavirus associated with severe nonsuppurative encephalomyelitis and sensory ganglionitis in sheep [9]. A recent study reported that a goat BooV was identified in a fecal sample in Hungary, closely related to the ovine BooV strain, and suggestive of the species *Boosepivirus C*, based on the species classification criteria [10]. Further surveillance of BooV C in goats revealed that 10 out of 62 samples were positive for BooV C specific PCR [6].

All the data presented in the literature show that *Boosepivirus B* is more common than *A* in cattle. In the present study, we report detection of two bovine BooV strains through next-generation sequencing in North America, and genomic characterization demonstrates that both strains belong to *Boosepivirus A*.

## 2. Materials and Methods

### 2.1. Samples

Two fecal samples were submitted to the University of Illinois Veterinary Diagnostic Laboratory (UI VDL) and Biovet Inc., Saint-Hyacinthe, QC, Canada.

### 2.2. RNA Extraction

At UI VDL, a fecal sample collected from a cow of unknown age was swabbed into 0.5 mL PBS buffer. Fecal PBS suspension solution was briefly vortexed for 15 s and centrifuged at 6800 rcf for 1 min. The nucleic acid sample was extracted from 100 microliters of supernatants of fecal suspension solution using the Qiagen BioSprint 96 One-For-All Vet Kit following the kit manual (Qiagen, Germantown, MD, USA). At Biovet, 1 g of a fecal sample collected from a young cow of unknown age was suspended with 5 mL PBS, and the nucleic acids were extracted using QIAamp DNA mini kit (Qiagen, Germantown, MD, USA).

### 2.3. Laboratory Testing

At UI VDL, molecular PCR tests for bovine coronavirus (BCoV), salmonella and rotavirus antigen testing (SA Scientific Ltd., San Antonio, TX, USA), and cryptosporidium acid fast stain were performed as requested by the client.

At Biovet, real-time PCR (RT-qPCR) tests were performed for the following agents: bovine viral diarrheal virus (BVDV), BCoV, bovine rotavirus A (BRV), *Bovine torovirus* (BToV) using Bovichek^®^ CIM-Virus (Biovet, St-Hyacinthe, QC, Canada), *Cryptosporidium* spp., *Giardia duodenalis*, *Salmonella* spp., ETEC-F5+ using Bovichek^®^ CIM-DNA (Biovet, Saint-Hyacinthe, QC, Canada), *Bovine astrovirus* (BAstV), *Bovine kobuvirus* (BKV), *Bovine nebovirus* (BNeV), and *Bovine norovirus* (BNoV) and *Bovine Boosepivirus* B (BooV B) using in-house assays.

### 2.4. Next-Generation Sequencing (NGS)

The extracted nucleic acids of both samples were subject to sequence-independent, single-primer amplification (SISPA) as previously described [11]. The nucleic acid was reverse-transcribed into cDNA using Superscript III (ThermoFisher, Waltham, MA, USA) and a random hexamer tagged with a known sequence, and then converted into dsDNA by Klenow polymerase (NEB, Ipswich, MA, USA), and further amplified using a single primer of the known sequence tag and Advantage 2 PCR kit (Takara Bio, Ann Arbor, MI, USA). The PCR products were purified using a QIAquick PCR Purification Kit (QIAGEN, Germantown, MD, USA) and quantified using Qubit broad-range and high-sensitivity kits (ThermoFisher, Waltham, MA, USA). The NGS library of each sample was prepared using Nextera XT kit (Illumina, San Diego, CA, USA) and sequenced on the Illumina MiSeq or iSeq 100 platform.

Two pairs of boosepivirus primers, the first pair for 5′ end UTR: 5F 5′-CCCCCTCCAATTCCCTT-3′ and boosepivirus-5R5′-CACAGGACACCCAAAGTAGTCGGT-3′ and the second pair for the region between VP1 and 2A: F3115 5′-AGGTTTGGTGCCCKAGACC-3′ and R3665 5′-ATCTCATCTGTTGCCTCAACTGTTAT-3′, were designed and used for amplifying the gaps of sequence. One-Step RT-PCR was performed using SuperScript™ III One-Step RT-PCR System with Platinum™ Taq DNA Polymerase (ThermoFisher, Waltham, MA, USA) and 5 µL RNA in a 25 µL reaction volume. Amplicon was purified using QIAquick PCR Purification Kit (QIAGEN, Germantown, MD, USA) and sequenced on MiSeq.

### 2.5. Bioinformatic Analysis

Raw FASTAQ data were assembled using SPAdes version v3.14.0 [12], and the assembled sequences were blasted against the NT blast database using the local NCBI blast command. Sequence alignment was performed using MAFFT online version 7 [13], sequence identity was calculated using BioEdit version 7.7.1, and a phylogenetic tree of Maximum Likelihood was constructed using MEGA version 7.0.26 [14]. Sequence mapping was performed using CLC Genomics Workbench version 20.0.4. The protein structure prediction of the capsid protein of bovine BooV was performed using the default setting of the online I-TASSER [15]. Sequences were deposited into the online GenBank database (PP035161 and PP035162).

## 3. Results

### 3.1. Routine Laboratory Testing Result

The fecal sample IL33712-22 at UI VDL was tested positive for BCoV (Ct value 20.0) and negative for rotavirus and Salmonella. No Cryptosporidium was detected through acid fast staining.

The fecal sample 1087562-21 was tested positive for BRV A (Ct value 18.8), BKV (Ct value 15.1), BNoV (Ct value 18.5) and BAstV (Ct value 20.8) and negative for BVDV, BCoV, BToV, BNeV, BooV B, *Cryptosporidium* spp., *Giardia duodenalis*, *Salmonella* spp., and ETEC-F5+.

### 3.2. NGS

Local blast of the assembled contigs for both IL33712-22 and 1087562-21 revealed the presence of bovine BooV, and mapping results showed that 167,561 (4.7%) and 839 (0.05%) reads mapped back to complete genomes bovine BooV IL33712-22 and 1087562-21, respectively. For the BooV1087562-21, two gaps (one gap in the 5′ end UTR, one gap between VP1 and 2A) were closed by amplicon-based sequencing.

Reads mapped to other viruses for IL33712-22 include 11,670 BCoV (0.33%), 1,114,715 BToV (31.30%), and 119,400 (3.35%) BNoV. Reads mapped to other viruses for 1087562-21 include 297 BKV 0.02%, 130 BAstV (0.01%), 15 BRV and 3 BNoV.

### 3.3. Genome Characterization of Boosepivirus

**Figure 1:**
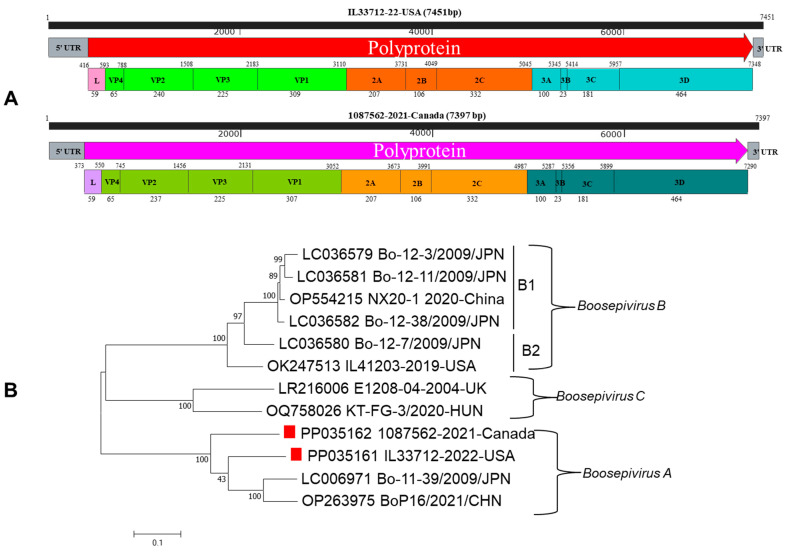
(**A**) Genomic diagram of bovine *Boosepivirus* IL33712-22 (GenBank accession number PP035161) with a genome size 7451 bp in length and bovine *Boosepivirus* 1087562-21 (GenBank accession number PP035162) with a genome size 7397 bp in length. Their genomes consist of 5′ and 3′ untranslated regions (UTRs) at both ends, and polyprotein in the middle encoding capsid proteins (VP4, VP2, VP3, and VP1) and nonstructural proteins 2A, 2B, 2C, 3A, 3B, 3C, and 3D. (**B**) Phylogenetic tree analysis of complete genomes of *Boosepivirus* A, B and C strains including two *Boosepivirus* A strains IL33712 and 1087562 from North America marked by a solid red square.

**Figure 2:**
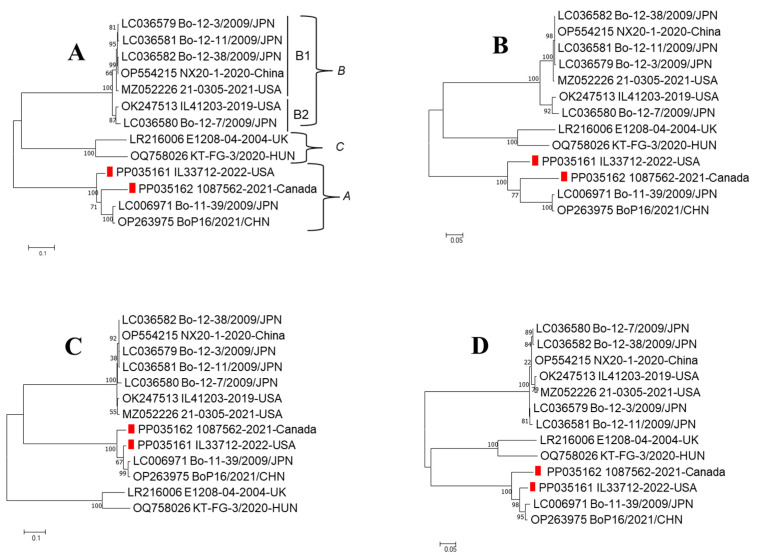
Phylogenetic tree analysis of amino acid sequences of polyprotein (**A**), P1 (**B**), P2 (**C**), and P3 (**D**). The trees were constructed via the MEGA 7.0.26 maximum likelihood method.

Online BLAST search (https://blast.ncbi.nlm.nih.gov/Blast.cgi?PROGRAM=blastn&BLAST_SPEC=GeoBlast&PAGE_TYPE=BlastSearch (accessed on 10 January 2024)) revealed that BooV IL33712-22 (GenBank accession #PP035161) had hits with only two BooV type A strains BoP16/2021/CHN (OP263975) and Bo-11-39/2009/JPN (LC006971) at the complete genome level (both 80% nucleotide identities). Similarly, the BooV 1087562-21 strain (GenBank accession #PP035162) also showed the highest sequence identities with these two strains (77% nucleotide identities) through BLAST. These data suggested that both BooV IL33712-22 and 1087562-21 might be *Boosepivirus A*. BooV IL33712-22 and 1087562-21 have characteristic genome organizations similar to those of other BooV members (Figure 1A). Phylogenetic tree analysis showed that IL33712-22 and 1087562-21 cluster together with the other two strains, Bo-11-39/2009/JPN and BoP16/2021/CHN, under the *Boosepivirus A* species at the complete nucleotide genome and amino acid of polyprotein, P1, P2, and P3 (Figure 1B and Figure 2A–D).

Further sequence identity analysis revealed that the US IL33712-22 strain had much higher identities in three BooV A strains at the complete nucleotide (75.0–77.6%) and polyprotein amino acid (86.1–90.4%) sequence levels, but very low identities (below 55%) in BooV B and C strains (Table 1). US IL33712-22 had lower identities in P1 (80.5–81.7%) than P2 (91.6–95.5%) and P3 (88.5–95.5%) in three BooV A strains. At the individual capsid and nonstructural protein levels, IL33712-22 had lower identities in three capsid proteins VP1 (77.6%), VP2 (80.0%), and VP3 (84.4%) but higher identities in all remaining proteins (90.3–100%) in BoP16/2021/CHN (Table 1).

Unlike the US BooV IL33712-22 strain, Canada 1087562-21 had relatively lower identities in the three other BooV A strains in the complete nucleotide genome (74–75%), polyprotein amino acid (85.8–86.1%), P1 (80.5–80.9%), P2 (89.7–91.6%), P3 (88.5–89.0%). 1087562-21 had lower identities in all four structural (<86.6%) and three nonstructural proteins (2A, 3A, 3C, <85.9%) in the other three BooV A strains and higher identities in the remaining four nonstructural proteins (2B, 2C, 3B, 3D) (up to 100%) (Table 1).

**Figure 3:**
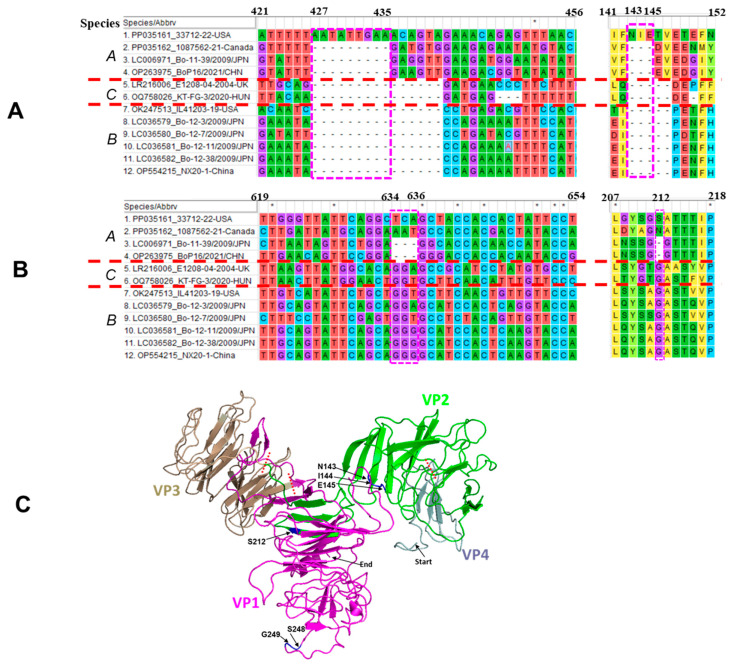
Nucleotide and amino acid sequence alignment of VP2 at two different regions, (**A**) 421–567 and (**B**) 619–654, for *Boosepivirus* A, B, and C strains. The regions containing insertions were marked with a red frame. The positions of nucleotides and amino acids are based on the IL33712-22 strain. (**C**) Structural modeling of capsid proteins of bovine *Boosepivirus* IL33712-22 strain carried out by I-TASSER (Iterative Threading ASSembly Refinement). VP4, VP2, VP3, and VP1 capsids were shown in pale cyan, green, wheat, and magenta colors, respectively. Three amino acid insertions at VP2 positions 143–145 NIE, one amino acid insertion at VP2 position 212 S, and two amino acid insertions at VP1 positions 248–249 SG are presented in blue.

**Figure 4:**
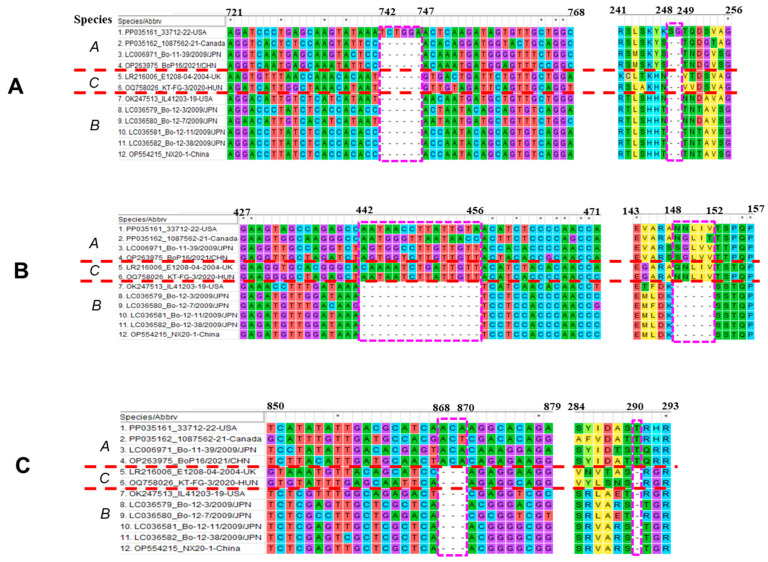
Nucleotide and amino acid sequence alignment of VP1 in three different regions, (**A**) 721–768, (**B**) 427–471 and (**C**) 850–879, for *Boosepivirus* A, B, and C strains. The regions containing insertions are marked with a red frame. The positions of nucleotides and amino acids are based on the IL33712-22 strain.

In the VP2, the US IL33712-22 strain has a 9-nt insertion at positions 427–435, resulting in an insertion of 3-amino acid (NIE) at positions 143–145 compared with three other BooV A strains (Figure 3A,C), and the two North American strains IL33712-22 and 1087562-21 have a 3-nt insertion at 634–636 and result in 1-aa (S/N) longer at position 212 compared with the two Asian BooV A Bo-11-39 and BoP16 strains (Figure 3B,C). In the VP1, the US IL33712-22 strain has a 6-nt insertion at positions 742–747, resulting in encoding 2-aa (SG) longer than the other three BooV A strains (Figure 4A).

Other than unique insertions in one or two North American BooV A strains, all types A and C have a 15-nt insertion at positions 442–456 and result in a 5-aa insertion at positions 148–152 compared with BooV B strains, and all type A strains have a 3-nt insertion at positions 868–870, resulting in 1-aa longer at position 290 in VP1 (Figure 4B,C).

## 4. Discussion

Picornaviruses causes different types of diseases in animals, including systemic (e.g., foot-and-mouth disease virus), neurological (e.g., porcine teschovirus), respiratory (e.g., equine rhinitis B virus), heart (e.g., encephalomyocarditis virus), and enteric (e.g., bovine kobuvirus) diseases [3,16,17,18,19,20]. Among the three BooV species, BooV A and B species originally identified in cattle with diarrhea might contribute to enteric diseases in cattle, whereas BooV C initially reported in sheep with encephalomyelitis could cause neurological disease in sheep [7,9]. Therefore, BooV might cause enteric and neurological diseases, and it still remains to be determined whether BooV is fulfilling Koch’s postulate causing diseases in cattle and sheep, depending on successful virus isolation through either conventional culture or reverse genetics. A recent study reported detection of a BooV C strain in a goat with diarrhea in Hungary, which was closely related to the ovine BooV strains [10]. In addition, a BooV B strain was detected in sheep in China [8]. These data suggest that BooV interspecies transmission or multiple tissue tropism could be possible.

Following initial identification, BooV B has been reported in the US and China [4,8]. Based on the information of deposited sequences at GenBank (e.g., accession #OR467533), BooV B was also present in South Korea. In the present study, we reported identification of two BooV A strains in North America through metagenomic sequencing. Both the IL33712-22 and 1087562-21 strains showed coinfection with other enteric pathogens. BooV A coinfection with other enteric pathogens might be similar to that of BooV B, which had higher coinfection rates (>50%). Before the present study for BooV A, there were no other studies reporting identification of BooV A in North America. Therefore, these data indicate that BooV A strains might be less common than BooV B strains. More efforts are needed to increase surveillance and characterization of BooV A in ruminants.

Genomic characterization of all BooV A strains demonstrated that two Asian strains (Bo-11-39/2009/JPN and BoP16/2021/CHN) shared very high sequence identities (86.7% and 98.3%) with each other and that the US IL33712-22 strain is phylogenetically more related to two Asian strains (>77.5% and >90.2%) than the Canada 1087562 strain (75% and 86.1%) at both complete nucleotide and polyprotein amino acid levels (Table 1). However, the Canada strain 1087562 shows similar identities to three other strains (74–75.0% of the complete nucleotide genome and 85.8–86.1% of polyprotein amino acid). The US strain IL33712-22 has 2-aa and 3-aa insertions at VP1 and VP2, respectively, compared with three other BooV A strains. Two North American strains have a 1-aa insertion in the VP2 compared with two Asian BooV A strains. The region containing a 3-aa insertion in VP2 of IL33712-12 is actually the area with a 2-aa insertion for all four BooV A strains compared with BooV B and C, and both BooV A and C have a 5-aa insertion in VP1 compared with BooV B. These insertions might contribute to viral pathogenesis and host adaptation, but their exact role remains to be determined.

In summary, our study is the first to report the detection of two BooV A strains in North America. Coinfection of two BooV A strains with other enteric pathogens is observed. Genomic characterization reveals the presence of strain-specific, North American-specific, species A-specific insertions. Our findings warrant continued surveillance and monitoring of BooV A in animals worldwide.

## Figures and Tables

**Table 1:** Sequence identities of bovine *Boosepivirus* strain IL33712-2022-USA (**A**), 1087562-2021-Canada (**B**), and BoP16/2021/CHN (**C**) with other *Boosepivirus* strains in different parts of the genome.

**A**																
PP035161_IL33712-2022-USA	Complete *	Polyprotein	P1	P2	P3	VP4	VP2	VP3	VP1	2A	2B	2C	3A	3B	3C	3D
OP263975_BoP16/2021/CHN	77.6	90.4	81.7	95.5	95.5	98.4	80.0	84.4	77.6	90.3	99.0	97.5	96.0	100	95.5	95.2
LC006971_Bo-11-39/2009/JPN	77.5	90.2	81.6	95.8	94.7	96.9	80.4	84.0	77.6	91.3	99.0	97.5	96.0	86.9	96.1	94.3
PP035162_1087562-2021-Canada	75.0	86.1	80.5	91.6	88.5	84.6	81.6	86.6	74.4	85.9	93.3	94.5	82.0	100	85.0	90.7
LR216006_E1208-04-2004-UK	54.8	52.4	56.8	42.1	59.8	64.6	54.7	60.3	55.3	29	38.8	51.1	46.0	52.1	64.8	61.0
OQ758026_KT-FG-3/2020-HUN	56.6	51.4	55.7	41.8	58.8	64.6	56.8	57.2	52.8	25.7	43.5	51.4	44.0	47.8	58.7	61.6
OK247513_IL41203-2019-USA	55.1	52.2	55.2	47.7	56.2	61.7	57.9	58.1	50.4	33.9	34.8	59.9	38.0	43.4	54.9	63.0
LC036579_Bo-12-3/2009/JPN	54.5	52.2	55.1	47.7	56.4	61.7	57.9	57.7	49.2	33.9	34.8	59.6	38.0	43.4	54.9	63.2
LC036580_Bo-12-7/2009/JPN	54.7	52.1	55.6	46.8	56.2	61.7	57.9	59.0	50.4	33.0	34.8	59.3	38.0	43.4	54.9	63.0
MZ052226_21-0305-USA	-	52.0	55.0	47.7	56.2	61.7	58.3	57.2	49.2	33.4	34.8	59.9	38.0	43.4	55.4	62.7
LC036581_Bo-12-11/2009/JPN	54.0	52.1	55.1	47.9	56.2	61.7	57.9	57.7	49.2	33.9	34.8	59.6	38.0	43.4	54.9	63.0
LC036582_Bo-12-38/2009/JPN	54.3	52.1	55.1	47.9	56.4	61.7	57.9	57.7	49.2	33.9	34.8	59.6	38.0	43.4	54.9	63.2
OP554215_NX20-1-2020-China	54.4	52.2	55.1	47.9	56.4	61.7	57.9	57.7	49.2	33.9	34.8	59.6	38.0	43.4	54.9	63.2
**B**																
PP035162_1087562-2021-Canada	Complete	Polyprotein	P1	P2	P3	VP4	VP2	VP3	VP1	2A	2B	2C	3A	3B	3C	3D
PP035161_IL33712-2022-USA	75.0	86.1	80.5	91.6	88.5	84.6	81.6	86.6	74.4	85.9	93.3	94.5	82.0	100	85.0	90.7
OP263975_BoP16/2021/CHN	74.1	86.1	80.9	90.2	89.0	84.6	81.8	85.7	75.8	84.0	92.4	93.3	82.0	100	84.5	91.8
LC006971_Bo-11-39/2009/JPN	74.0	85.8	80.8	89.7	88.8	86.1	81.8	85.3	75.5	82.6	92.4	93.3	84.0	86.9	85.6	91.1
LR216006_E1208-04-2004-UK	53.6	51.2	54.4	41.7	59.5	60.0	52.9	62.1	50.1	28.5	38.8	50.8	45.0	52.1	65.3	60.3
OQ758026_KT-FG-3/2020-HUN	55.1	50.7	54.2	42.1	57.9	61.5	55.0	60.3	49.3	25.2	42.5	53.2	41.0	47.8	59.3	60.5
OK247513_IL41203-2019-USA	53.2	51.2	53.0	47.4	56.0	57.3	55.2	59.4	46.6	32.0	33.9	60.5	38.0	43.4	58.7	61.0
LC036579_Bo-12-3/2009/JPN	53.6	51.4	53.4	47.7	56.1	57.3	55.6	58.5	47.5	33.0	33.9	60.2	37.3	43.4	58.7	61.5
LC036580_Bo-12-7/2009/JPN	53.4	51.3	53.1	47.1	56	57.3	54.8	59.0	47.2	33.0	33.9	60.5	37.3	43.4	58.7	61.2
MZ052226_21-0305-USA	-	51.1	53.1	47.4	55.9	57.3	56.1	58.1	46.6	31.6	33.9	60.5	37.3	43.4	59.3	60.8
LC036581_Bo-12-11/2009/JPN	53.0	51.3	53.3	47.6	56.0	57.3	55.6	58.5	47.2	33.0	33.9	60.2	37.3	43.4	58.2	61.5
LC036582_Bo-12-38/2009/JPN	53.3	51.3	53.3	47.6	56.0	57.3	55.6	58.5	47.2	33.0	33.9	60.2	37.3	43.4	58.7	61.2
OP554215_NX20-1-2020-China	53.5	51.3	53.3	47.6	56.1	56.7	55.6	58.5	47.2	33.0	33.9	60.2	37.3	43.4	58.7	61.5
**C**																
OP263975_BoP16/2021/CHN	Complete	Polyprotein	P1	P2	P3	VP4	VP2	VP3	VP1	2A	2B	2C	3A	3B	3C	3D
LC006971_Bo-11-39/2009/JPN	86.7	98.3	99.0	98.1	97.9	98.4	99.5	99.5	98.3	94.2	100	100	98.0	86.9	97.2	98.7
PP035161_IL33712-2022-USA	77.6	90.4	81.7	95.5	95.5	98.4	80.0	84.4	77.6	90.3	99.0	97.5	96.0	100	95.5	95.2
PP035162_1087562-2021-Canada	74.1	86.1	80.9	90.2	89.0	84.6	81.8	85.7	75.8	84.0	92.4	93.3	82.0	100	84.5	91.8
LR216006_E1208-04-2004-UK	56.0	52.1	55.5	41.8	60.7	64.6	54.6	62.9	49.8	28.5	37.9	51.4	46.0	52.1	65.3	62.3
OQ758026_KT-FG-3/2020-HUN	56.2	51.1	55.1	40.9	59.1	64.6	54.6	59.9	50.6	25.2	42.5	50.8	44.0	47.8	57.6	62.7
OK247513_IL41203-2019-USA	54.5	51.6	54.6	47.3	55.6	61.7	54.4	59.4	50.4	31.6	35.7	60.5	36.5	43.4	54.9	62.3
LC036579_Bo-12-3/2009/JPN	54.5	51.6	54.3	47.6	55.6	61.7	54.8	59.4	48.5	32.5	35.7	60.2	36.5	43.4	54.9	62.3
LC036580_Bo-12-7/2009/JPN	54.9	51.8	55.2	46.5	55.5	61.7	55.2	59.0	51.7	31.6	35.7	59.9	36.5	43.4	54.9	62.1
MZ052226_21-0305-USA	-	51.5	54.3	47.3	55.6	61.7	54.8	59.4	48.8	31.1	35.7	60.5	36.5	43.4	55.4	62.1
LC036581_Bo-12-11/2009/JPN	54.0	51.7	54.5	47.4	55.6	61.7	54.8	59.4	48.8	32.5	35.7	60.2	36.5	43.4	54.9	62.3
LC036582_Bo-12-38/2009/JPN	54.3	51.6	54.5	47.4	55.6	61.7	54.8	59.4	48.8	32.5	35.7	60.2	36.5	43.4	54.9	62.3
OP554215_NX20-1-2020-China	54.4	51.7	54.5	47.4	55.6	61.7	54.8	59.4	48.8	32.5	35.7	60.2	36.5	43.4	54.9	62.3

* Values represent % identity of complete genome at nucleotide level. All other % identities are at the protein amino acid levels; -: not available.

## Data Availability

The data presented in this study are deposited into GenBank (GenBank accession number PP035161 and PP035162).
